# Editorial: Automation and artificial intelligence in radiation oncology

**DOI:** 10.3389/fonc.2022.1038834

**Published:** 2022-10-12

**Authors:** Savino Cilla, Jose Eduardo Villarreal Barajas

**Affiliations:** ^1^ Medical Physics Unit, Gemelli Molise Hospital, Campobasso, Italy; ^2^ Royal Devon University Healthcare Medical Physics Department, Royal Devon and Exeter Hospital, Exeter, United Kingdom

**Keywords:** artificial intelligence (AI), automation, machine learning, radiomics, radiation oncology

The ongoing advancement of radiation oncology has always been significantly influenced by technology. A number of therapeutic advancements have been made recently as a result of technology-driven advancements in radiotherapy planning and delivery. In particular Particularly in the field of radiation oncology, artificial intelligence approaches are spreading more widely and moving from the realm of specialized research to that of accepted clinical practice. Automation and big data analysis have drawn a new era of treating cancer patients with precision and outcome prediction. The continues increase of computing power together with the improvement of treatment accuracy in fighting cancers could lead to huge progress in increasing patient’s outcomes and survival rate. The integration of artificial intelligence with modern radiation therapy technologies has the potential to herald an unprecedented change for the field of radiation oncology.

The aim of this Topic was to collate original researches focusing on new developments in the application of machine learning and deep learning processes, patient outcome prediction, treatment technique improvements with automation and applications of radiomics, an emerging and promising research field based on quantitative imaging technology in the radiation oncology field. All these aspects have been well-captured in the present Research Topic which has been successfully launched in Frontiers in Oncology. We were thrilled to get a large number of contributions from authors of their most recent research findings on automation and artificial intelligence techniques for radiation oncology purposes. Twenty-one papers were finally accepted after rigorous reviews for a total of 177 authors. Contributions came from various nations and regions, including China, Italy, South Korea, Thailand, the United States and Indonesia.

Several researchers investigated the possibility for automated treatment planning solutions generated by AI algorithms to improve quality, decrease variability, and shorten planning times. One of the most time-consuming step of radiation therapy is the manual segmentation of target and normal structures, which is subject to high intra- and inter-observer variability. Recently, numerous research groups have been focusing on the use of AI to increase the speed and accuracy in the definition of clinical target volumes for treatment. Convolutional neural network-based deep learning models have made significant advancements and demonstrated major promise as tools for automated segmentation of target volumes and organs-at-risk (OARs). For CTV delineation in cervical cancer, Liu et al. suggested a novel adversarial deep-learning-based auto-segmentation algorithm. To directly test the model and reduce inter- and intra-observer variability, a three-stage multicenter randomized controlled evaluation procedure was created. The evaluated AI model was shown to be precise and on par with the manual CTV segmentation in patients with cervical cancer. By integrating the fully convolutional network (FCN) and atrous convolution deep learning techniques, Xie et al. sought to fully automate the organs segmentation. According to the authors, this network model may efficiently increase the precision of automated segmentation of chest computed tomography images in thoracic radiotherapy. To successfully avoid radiation side effects, precise target volume and OAR delineation is essential in head-neck malignancies. In order to segment the thyroid gland on localized CT scans and to identify the gland as an OAR in radiotherapy, Wen et al. devised a model that incorporated a Spatial Squeeze and Channel Excitation Block (cSE) attention mechanism with HRNet. Due to the low contrast at the tumor’s border and the wide range of tumor sizes and morphologies between different stages, the delineation of target volumes in nasopharyngeal cancer is a particularly difficult process. To solve the aforementioned issues, Yang et al. proposed a new three-dimensional (3D) automatic segmentation system that uses cascaded multiscale local augmentation of convolutional neural networks. The suggested approach may enhance and facilitate clinical applications by precisely segmenting NPC in CT scans from multi-institutional datasets. The possibility of using deep learning to automatically delineate multiple contours for breast cancer radiation therapy was examined by Dai et al. Their study showed that the devolped deep learning techniques can reliably produce target and OAR contours on planning CT and daily synthetic CT images from CBCT images, which may significantly speed up the re-planning process and satisfy the needs of online plan adaptation.

The goal of radiotherapy plan optimization is to find the optimal balance between two competing goals: delivering the highest radiation dose to the target while delivering the lowest radiation dose to nearby OARs. These OARs are typically given a numerical weight to reflect their relative importance in the optimization calculus. In order to produce a plan that fulfills the minimal acceptable threshold for each aim, physicists must repeatedly adjust the parameters that control radiation dose deposition. This fine-tuning typically goes on until time resources run out, at which point the planner is compelled to decide on the best plan he can achieve. Therefore, plan quality may strongly vary between planners and between clinical institutions. Additionally, the time and labor requirements of the existing planning paradigm can put patients at risk for delays and potentially suboptimal care while also appearing to be insurmountable barriers to adaptive radiotherapy. In this perspective, the introduction of automated systems may translate in important benefits as time saving, high quality planning, and protocol standardization, as reported by Cagni et al. Together with template-based iterative planning (1), the use of knowledge-based automated planning (KBP) techniques has recently received a special attention. Using machine learning techniques that learn from databases of previous clinically acceptable plans, KBP may assist physicists and radiation oncologists to find the best solutions for planning optimization. Castriconi et al. implemented a KBP solution for right and left-sided whole breast treatment through a new volumetric technique mimicking conventional tangential fields irradiation that can efficiently replace manually optimized plans. Xu et al. evaluated the effectiveness of a proton-specific KBP model in the development of robustly optimized intensity-modulated proton therapy plans for the treatment of advanced head and neck cancer patients, reporting that the quality of KBP plans is comparable to, and occasionally even exceeds, that of the expert plans. In this clinical setting, radiation therapy is the primary therapeutic option for early and locally progressed nasopharyngeal cancer. When compared to computed tomography, magnetic resonance imaging (MRI) has the advantage of high soft-tissue resolution, but it does not provide information on electron density (ED) for planning radiotherapy. To provide the necessary ED data for MRI-only planning, Ma et al. created a pseudo-CT generating approach. The suggested deep learning model can precisely predict CT from MRI, and the resulting pCT can be used in accurate dose estimations.

Proton therapy may also greatly benefit of using AI strategies and techniques. For example, a great interest in beam angle optimization research has been on the rise recently for proton therapy, in order to generate optimal proton plan. Cheon et al. suggested a method for beam angle optimization based on a convolutional neural network to automatically find the optimal beam angles for proton treatments set with the double-scattering delivery approach (BAODS-Net). This approach dramatically reduced the planning time increasing the potential for a real-time adaptive proton radiotherapy. Furthermore, it is well known that a double scattering proton system’s beam output fluctuates depending on the beam option, range, and modulation, translating in inaccurate modeling by the treatment planning system. Because of this, the majority of proton centers with a double scattering beam system must measure the output of patient-specific proton beams in a water phantom in order to determine the necessary machine output. Three machine learning algorithms were developed by Zhu et al. to efficiently estimate the output of a proton beam using a Gaussian process regression model with various kernels. One of these models showed accurate estimation, meeting the ±3% clinical requirement.

A second fundamental point is the growing application of artificial intelligence techniques for prediction purposes.

First, methods for machine learning have been investigated, with an emphasis on applications for machine and patient-specific quality assurance (QA) (2). The performance of various delivery system components, such as the multileaf collimator (MLC), imaging system, mechanical parameters, and dosimetric parameters, can be examined using machine learning. As a result, a “virtual” QA may forecast passing rates using different measurement techniques, different treatment planning systems, and different treatment delivery machines across multiple institutions. In this topic, Huang et al. introduced a new QA prediction model based on UNet++ using the dose distribution as input. This model was able to predict the gamma-pass rates for various gamma criteria as well as provide classification results.

Secondly, AI models have recently demonstrated the potential for effective toxicity prediction aiming to limit radiotherapy-related side effects (3). The proactive, rather than reactive, management of acute and late toxicities in patients is exacerbated by the mostly unpredictable occurrence and/or intensity of such side effects. Nevertheless, it is possible to create predictive models of radiation toxicities based on imaging data and risk variables, such as specific clinical traits, germline genetic alterations, and the radiation dose distributions, and these models can be used to guide treatment planning. Additionally, multi-omic data may capture complex tumor features, contributing to a comprehensive patient risk assessment. In particular, two complementary strategies have emerged in recent years: the integration of patient-specific biological risk factors into dose–volume-based outcome models (called radiogenomics), and the integration of imaging together with treatment-related and biological data for outcomes prediction (called radiomics). Both these approaches have the potential to develop personalized and tailored treatment plans.

The current advances and challenges in radiomics of brain tumors have been highlighted by Yi et al. The authors demonstrate how radiomics, in contrast to conventional brain imaging, offers quantitative data related to important biologic characteristics and application of deep learning which sheds light on the complete automation of imaging diagnosis.

In patients with ovarian cancer, Yu et al. assessed the accuracy of radiomics characteristics based on multiparameter magnetic resonance imaging for peritoneal carcinomatosis. A multi-factor logistic regression method was utilized to create a radiomics nomogram in combination with radiomics features and clinicopathological risk factors, reporting a better diagnostic effect than the clinical model, able to identify peritoneal carcinomatosis in ovarian cancer patients before surgery.

Gastric cancer is a typical heterogeneous malignant tumor. Chemotherapy is ineffective against this tumor and this is a common cause of tumor recurrence and metastasis. Conventional pathological TNM prediction focuses on cancer cells to predict prognosis, but they do not provide adequate prediction. Jin et al. devoloped a radiomics signature in order to predict patients’ overall survival and disease-free survival after undergoing surgery for gastric cancer. The radiomics trait-associated genes identified clinically significant biological pathways and possible drug metabolic mechanisms for chemotherapy agents.

With respect to rectal cancer, although several prognosis nomograms have been established, statistical tools for predicting long-term survival in rectal cancer are lacking. Additionally, neither qualitative nor quantitative imaging findings were included in modern prognostic analyses. Nie et al. used a radiomics signatures and multiparametric MRI data to build a predictive model able to predict 5-year overall survival for patients with advanced rectal cancer. An interesting aspect of the detected radiomics signature was that it contained three from dynamic contrast-enhanced (DCE)-MRI, four from anatomical MRI, and one from functional diffusion-weighted imaging (DWI). This brought attention to how crucial multiparametric MRI is in addressing the problem of estimating long-term survival in rectal cancer.

The 5-year survival rate of lung cancer is significantly increased by early detection and treatment. Immunotherapy has recently grown quickly, caught the attention of more and more oncologists, and established itself as a significant area of study in the field of tumor therapy. The immunotherapy against programmed cell death protein 1 (programmed death-1, PD-1) and its ligand 1 (programmed death ligand-1, PD-L1) has been used in non-small cell lung cancer (NSCLC), and good results have been achieved in patients, especially in individuals with high expression of PD-L1 (4). Finding a fresh method to gauge PD-L1 expression level is thus critically required. Based on this premises, Li et al. aimed to evaluate the expression of PD-L1 in patients with NSCLC by radiomic features of 18F-FDG PET/CT and clinicopathological characteristics. In order to predict PD-L1 expression in individual NSCLC patients, the authors generated a prediction model that used both the radiomic signature and clinicopathologic risk variables. Significant correlations were found between the radiomic signature and PD-L1 expression in lung cancers. The aforementioned papers reported how the recently emerged radiomics methods are able to extract a large number of spatial features from medical images in order to predict therapeutic responses. Enlightened by those works, a recent approach called “dosiomics” has been put out, in an effort to extract spatial features from dose distribution for radiotherapy response prediction. It has been shown that the dosiomics features may be able to improve radiation therapy toxicity prediction since they have more dose distribution data than DVH features (5). For example, dosiomics informations can be used for the prediction of radiation pneumonitis. Puttanawarut et al. investigated the feasibility of of dosiomics and radiomics features to predict the development of radiation pneumonitis over traditional dose-volume histogram. Then, four predictive models for radiation pneumonitis were compared on a esophageal and a lung cancer datasets, resulting in predictive performance of the dosiomics- and radiomics-based models significantly higher than that of the DVH-based model.

Finally, it must be underlined that studies on the dosiomics and radiomics features are still in the early stage and yet there exist some concerns regarding the stability and generalizability of this texture analysis. For example, it has been reported that some dosiomics features are unstable across various grid resolutions or dose calculation algorithms (6), showing that the reproducibility of dosiomics features depends on the process of producing images. Moreover, for many cancers, because of inter-fractional error, a different total number of fractions may induce different error behavior. These errors may also further affect the reproducibility of dosiomics features. Puttanawarut et al. investigated the stability of dosiomics features under random inter-fractional error and evaluated the uncertainties in the values of dosiomic features under inter-fractional error with IMRT and VMAT in a lung cancer dataset. The authors reported that some dosiomics features were found not reliable under inter-fractional error and with lower fraction numbers.

## Conclusions

The ultimate goal of this Research Topic was to promote research and development of automation, advanced computing and AI applications in radiation oncology by publishing high-quality research articles. The 21 papers published in this Research Topic reported promising results and offered new and original perspectives regarding the role of AI in radiation oncology. We thank all the authors of the published papers for their valuable contributions and the referees for their rigorous review.

## Author contributions

Both authors have made a substantial, direct, and intellectual contribution to the work and approved it for publication.

## Conflict of interest

The authors declare that the research was conducted in the absence of any commercial or financial relationships that could be construed as a potential conflict of interest.

## Publisher’s note

All claims expressed in this article are solely those of the authors and do not necessarily represent those of their affiliated organizations, or those of the publisher, the editors and the reviewers. Any product that may be evaluated in this article, or claim that may be made by its manufacturer, is not guaranteed or endorsed by the publisher.
